# Bile Acid Sequestrant, Sevelamer Ameliorates Hepatic Fibrosis with Reduced Overload of Endogenous Lipopolysaccharide in Experimental Nonalcoholic Steatohepatitis

**DOI:** 10.3390/microorganisms8060925

**Published:** 2020-06-19

**Authors:** Yuki Tsuji, Kosuke Kaji, Mitsuteru Kitade, Daisuke Kaya, Koh Kitagawa, Takahiro Ozutsumi, Yukihisa Fujinaga, Hiroaki Takaya, Hideto Kawaratani, Tadashi Namisaki, Kei Moriya, Takemi Akahane, Hitoshi Yoshiji

**Affiliations:** Department of Gastroenterology, Nara Medical University, Kashihara, Nara 634-8521, Japan; tsujih@naramed-u.ac.jp (Y.T.); Kitadem@naramed-u.ac.jp (M.K.); kayad@naramed-u.ac.jp (D.K.); Kitagawa@naramed-u.ac.jp (K.K.); ozutaka@naramed-u.ac.jp (T.O.); fujinaga@naramed-u.ac.jp (Y.F.); htky@naramed-u.ac.jp (H.T.); kawara@naramed-u.ac.jp (H.K.); tadashin@naramed-u.ac.jp (T.N.); moriyak@naramed-u.ac.jp (K.M.); stakemi@naramed-u.ac.jp (T.A.); yoshijih@naramed-u.ac.jp (H.Y.)

**Keywords:** nonalcoholic steatohepatitis, sevelamer, lipopolysaccharide, toll-like receptor 4

## Abstract

Despite the use of various pharmacotherapeutic strategies, fibrosis due to nonalcoholic steatohepatitis (NASH) remains an unsatisfied clinical issue. We investigated the effect of sevelamer, a hydrophilic bile acid sequestrant, on hepatic fibrosis in a murine NASH model. Male C57BL/6J mice were fed a choline-deficient, L-amino acid-defined, high-fat (CDHF) diet for 12 weeks with or without orally administered sevelamer hydrochloride (2% per diet weight). Histological and biochemical analyses revealed that sevelamer prevented hepatic steatosis, macrophage infiltration, and pericellular fibrosis in CDHF-fed mice. Sevelamer reduced the portal levels of total bile acid and inhibited both hepatic and intestinal farnesoid X receptor activation. Gut microbiome analysis demonstrated that sevelamer improved a lower α-diversity and prevented decreases in *Lactobacillaceae* and *Clostridiaceae* as well as increases in *Desulfovibrionaceae* and *Enterobacteriaceae* in the CDHF-fed mice. Additionally, sevelamer bound to lipopolysaccharide (LPS) in the intestinal lumen and promoted its fecal excretion. Consequently, the sevelamer treatment restored the tight intestinal junction proteins and reduced the portal LPS levels, leading to the suppression of hepatic toll-like receptor 4 signaling pathway. Furthermore, sevelamer inhibited the LPS-mediated induction of fibrogenic activity in human hepatic stellate cells in vitro. Collectively, sevelamer inhibited the development of murine steatohepatitis by reducing hepatic LPS overload.

## 1. Introduction

Nonalcoholic fatty liver disease (NAFLD), which encompasses a clinicopathological spectrum of liver diseases ranging from simple steatosis to nonalcoholic steatohepatitis (NASH), fibrosis, and cirrhosis, is defined as ectopic lipid accumulation in more than 5% of hepatocytes in the absence of other secondary causes such as chronic viral hepatitis, excessive alcohol consumption, autoimmune hepatitis, and congenital hepatic disorders [1,2,3]. NASH is pathologically characterized by the presence of hepatic steatosis, hepatocellular damage, and the hepatic infiltration of macrophages, leading to progressive hepatic fibrosis [4]. Recent clinical evidence suggests that significant fibrosis is the most critical histological feature associated with overall mortality, liver transplantation, and liver-related events in patients with NASH [5]. The several pharmacological strategies which have been proposed for the treatment of NASH-based fibrosis will not be ready for clinical use in near future [6,7]. NASH pathogenesis has been recognized to be based on “multiple parallel hits” [4]. Notably, a NASH pathological state often triggers a derangement in the gut–liver axis that involves intestinal dysbiosis and intestinal barrier dysfunction [8]. In particular, lipopolysaccharide (LPS), a cell wall component of intestinal gram-negative bacteria, is a canonical ligand for Toll-like receptor 4 (TLR4) signaling in promotion of liver fibrosis [9,10,11]. In fact, bacterial overgrowth and intestinal hyperpermeability are common in patients with NASH and LPS is a major inducer of neutrophil and macrophage infiltration of the liver of patients with NASH [12,13]. We have recently shown that both endogenous and exogenous LPS overload worsens hepatic steatosis, inflammation, and fibrosis through the LPS/TLR4 pathway in rodent models [9,14]. Moreover, bile acids have antibacterial properties [15]. Bile acid has shown to promote digestion and absorption of fat-soluble foods and improve endotoxemia to preserve the intestinal barrier [16,17]. The administration of antibiotics reduced the development of NAFLD and altered the composition of bile acid and the suppression of signaling by farnesoid X receptor (FXR) [18]. Thus, the interaction between intestinal bacteria and bile acid has provided good evidence for gut microbiota-targeted therapy for NAFLD.

Bile acid sequestrants (BASs), which are indicated for the management of hypercholesterolemia, exert their activity by luminal entrapment of bile acids in the gastrointestinal tract and their removal from the enterohepatic circulation, with a subsequent compensatory augmentation of low-density lipoprotein clearance and conversion of cholesterol to bile acids [19,20]. In addition to their lipid-lowering activities, BASs have been reported to exert an antidiabetic effect [21]. Possible mechanisms include the involvement of FXR and Takeda G-protein receptor 5 within the intestines as well as the involvement of FXR in the liver, which might eventually lead to the downregulation of endogenous glucose production [22]. Moreover, BASs might stimulate the systemic release of incretin hormones, specifically glucagon-like peptide-1 and glucose-dependent insulinotropic polypeptide [22,23,24]. Based on these effects, recent studies further demonstrated that BASs could improve obesity and hepatic steatosis in human and rodent models [25,26,27].

Sevelamer is a hydrophilic, nonabsorbable, amine-based resin BAS approved for the treatment of hyperphosphatemia in patients with chronic kidney disease [28]. Similar to other BASs, sevelamer was found to improve steatohepatitis by counteracting the dysregulation of hepatic and intestinal FXR signaling in Western diet (WD)-fed mice [29]. Several recent reports demonstrated that sevelamer might possess anti-inflammatory properties through increased binding affinity with bacterial LPS. Hauser et al. showed that sevelamer significantly improved systemic inflammation and endotoxemia in a rat model of chronic renal failure [30], whereas Navarro-Gonzalez et al. reported that patients on hemodialysis who were treated with sevelamer exhibited lower serum levels of high-sensitivity C-reactive protein, interleukin (IL)-6, endotoxins, and soluble CD14 [31]. However, few studies have focused on whether sevelamer-mediated improvement of endotoxemia is involved in its effect on the development of NASH-based hepatic fibrosis.

The present study aimed to elucidate the effect of sevelamer in improving the pathology of NASH-based fibrosis and determine the therapeutic mechanisms associated with reduced hepatic overload of LPS by gut microbial modification and intestinal LPS adsorption in addition to its capacity of bile acid sequestration using a mouse model of NASH.

## 2. Materials and Methods

### 2.1. Animals and Experimental Protocol

Eight-week old male C57BL/6J mice (CLEA Japan, Osaka, Japan) were divided into the following four diet groups: (1) choline-sufficient amino acid containing normal fat (CSNF) diet (research diets, New Brunswick, NJ, USA) with vehicle (*n* = 10), (2) CSNF diet with sevelamer hydrochloride (Chugai Pharmaceutical, Tokyo, Japan) (*n* = 10). (3) choline-deficient, L-amino acid-defined (CDAA) diet containing high-fat (CDHF) (research diets) with a vehicle (*n* = 10), and (4) the CDHF diet with sevelamer hydrochloride (*n* = 10). The components of CSNF and CDHF were shown in Supplementary Table S1. Sevelamer hydrochloride were administered as mixture (2% per diet weight) as described previously, and the same amount of lactose hydrate was used as vehicle [29]. All mice were housed in stainless steel mesh cages under controlled conditions (23 °C ± 3 °C with a relative humidity of 50% ± 20%, 10–15 air changes/hour, and 12 h of light/day). All animals were allowed access to tap water ad libitum throughout the experimental period. All mice were sacrificed after 12 weeks of experimental breeding. At the end of the experiment, all mice underwent the following procedures: anesthesia, opening of the abdominal cavity, blood collection via puncture of the aorta and portal vein, collection of fecal samples for microbiome analysis and measurement of LPS levels, and harvesting of liver and terminal ileum for histological and molecular evaluation. Serum biological markers were assessed by routine laboratory methods. All animal procedures were performed in compliance with the recommendations of the Guide for Care and Use of Laboratory Animals (National Research Council), and the study was approved by the animal facility committee of Nara Medical University (authorization number: 12305).

### 2.2. Cell Culture

LX-2 human stellate cells (HSCs) were purchased from the Japanese Cancer Research Resources Bank (Tokyo, Japan). The cells were maintained as monolayer cultures in Dulbecco’s modified Eagle medium supplemented with 10% fetal bovine serum and 1% penicillin/streptomycin in an incubator at 37 °C and 5% CO_2_. For all assays, cells were pre-incubated with different concentrations of LPS (O55:B5) (100 ng/mL; Sigma-Aldrich, St. Louis, MO, USA) and/or sevelamer hydrochloride (0, 2, 5, 10, 25, or 50 mg/mL) for 24 h and with 5 ng/mL recombinant human transforming growth factor-β1 (TGF-β1) (catalog number: T7039) for 6 h (Sigma-Aldrich).

### 2.3. Histological and Immunohistochemical Analyses

The liver sections were fixed with 10% formalin and embedded in paraffin. Subsequently, 5-µm-thick paraffin-embedded sections were stained with hematoxylin and eosin. Liver steatosis and inflammation were evaluated based on a previously described scoring system [32]. The liver sections were also stained with Sirius Red. For immunohistochemistry, the sections were pre-treated using heat mediated antigen retrieval with sodium citrate buffer (pH6.0) for 20 min. As the primary antibodies, alpha-smooth muscle actin (α-SMA) (ab5494; 1:200 dilution, Abcam, Cambridge, UK) and F4/80 (ab100790; 1:100 dilution, Abcam) were used with staining performed according to the suppliers’ recommendations. A goat anti-rabbit biotinylated secondary antibody was used to detect the primary, and visualized using an HRP conjugated ABC system (Vector Laboratories, Burlingame, CA, USA). DAB was used as the chromogen. Specimens for histology and immunohistochemistry were observed under an optical microscope (BX53; OLYMPUS, Tokyo, Japan) equipped with a digital microscope camera (DP20-5; OLYMPUS). The National Institutes of Health (NIH) ImageJ software (http://imagej.nih.gov/ij/) was used for quantitative analyses. All quantitative analyses were performed for 5 fields per each section in high-power fields (HPFs) at 400-fold magnification.

### 2.4. Measurement of Total Bile Acid Levels

Serum total bile acid levels in the portal vein were measured by Mouse Total Bile Acid Assay Kit (Crystal Chem, Elk Grove Village, IL, USA), according to the manufacturer’s instructions.

### 2.5. Measurement of LPS Levels

LPS concentrations in murine portal vein serum samples, murine colon fecal samples, and in vitro cultured media were measured by the *ToxinSensor chromogenic LAL endotoxin assay kit (Genscript, Piscataway, NJ, USA),* according to the manufacturer’s instructions. LPS levels were expressed as endotoxin units.

### 2.6. RNA Extraction and Reverse Transcription-Quantitative Polymerase Chain Reaction

Total RNA was extracted from the frozen liver and terminal ileum tissue samples as well as from whole cell lysates using the RNeasy mini kit (Qiagen, Tokyo, Japan) per the manufacturer’s instructions. Next, total RNA was reverse transcribed into complementary DNA (cDNA) using the High-Capacity RNA-to-cDNA kit (Applied Biosystems, Foster City, CA, USA), according to the manufacturer’s instructions. Reverse transcription-quantitative polymerase chain reaction of the cDNA with gene-specific primer pairs (Table 1) was performed using StepOnePlus Real-time PCR system and SYBR green from Applied Biosystems (Applied Biosystems). Relative gene expression levels were determined using 18s ribosomal RNA as the internal control. We treated RNA samples with DNAse in order to remove DNA contamination with TURBO DNA-free™ DNase (Invitrogen). Relative amount of target mRNA per cycle was determined by applying a threshold cycle to the standard curve. All reactions were performed using a 1:10 diluted cDNA while mRNA expression levels were estimated using the 2ΔΔCT method and showed relative values to each control group.

### 2.7. Protein Extraction and Western Blotting

Whole cell lysates were prepared from 200 mg frozen ileal tissue using Tissue-Protein Extraction Reagent (T-PER) supplemented with proteinase and phosphatase inhibitors (all from Thermo Scientific, Waltham, MA, USA). In total, 50 μg whole cell lysates were separated by sodium dodecyl sulfate-polyacrylamide gel electrophoresis (NuPAGE; Thermo Fisher Scientific) and transferred to Invitrolon polyvinylidene difluoride membranes (Thermo Fisher Scientific), which were subsequently blocked for one hour with 5% bovine serum albumin in Tris-buffered saline supplemented with Tween-20. The membranes were then incubated overnight with antibodies against ZO-1 (#61-7300; 1:250 dilution, Invitrogen, Carlsbad, CA, USA), occludin (ab168986; 1:1000 dilution Abcam), claudin-4 (ab15104; 1:1000 dilution, Abcam), and β-actin (#4967; 1:10000 dilution, Cell Signaling Technology, Danvers, MA, USA). The membranes were washed and incubated with Amersham ECL horseradish peroxidase-conjugated immunoglobulin G F(ab)2 fragment antibody (1:5000 dilution; GE Healthcare Life Sciences, Piscataway, NJ, USA) and developed using Clarity Western enhanced chemiluminescence substrate (BioRad, Hercules, CA, USA).

### 2.8. Analysis of Gut Microbiota

The fecal samples from the colon of five mice in each experimental group at the end of experiments were stored at −80 °C before analysis by next generation sequencing performed by Cykinso (Tokyo, Japan). DNA extraction from the fecal samples was performed using an automated DNA extraction machine (GENE PREP STAR PI-480, Kurabo Industries Ltd., Osaka, Japan) according to the manufacturer’s protocol. The V1–V2 region of the 16S rRNA gene was amplified using forward primer (16S_27Fmod: TCG TCG GCA GCG TCA GAT GTG TAT AAG AGA CAG AGR GTT TGA TYM TGG CTC AG) and reverse primer (16S_338R: GTC TCG TGG GCT CGG AGA TGT GTA TAA GAG ACA GTG CTG CCT CCC GTA GGA GT) with KAPA HiFi Hot Start Ready Mix (Roche, Basel, Switzerland). To sequence 16S amplicons by Illumina MiSeq platform, dual index adapters were attached using the Nextera XT Index kit (Illumina, San Diego, CA, USA). Each library was diluted to 5 ng/µL, and equal volumes of the libraries were mixed to 4 nM. The DNA concentration of the mixed libraries was quantified by qPCR with KAPA SYBR FAST qPCR Master mix (KK4601, KAPA Biosystems) using primer 1 (AAT GAT ACG GCG ACC ACC) and primer 2 (CAA GCA GAA GAC GGC ATA CGA). The library preparations were carried out according to 16S library preparation protocol of Illumina (Illumina). Libraries were sequenced using the MiSeq Reagent Kit v2 (500 Cycles), with 250 bp paired-end reads. The paired-end reads of the partial 16S rRNA gene sequences were clustered by 99% nucleotide identity, and then assigned taxonomic information using Greengenes [33] through Quantitative Insights Into Microbial Ecology (QIIME) 2 pipeline (version 2019.4) [34]. The steps for data processing and assignment based on the QIIME 2 pipeline were as follows: (1) Divisive Amplicon Denoising Algorithm (DADA) 2 for joining paired-end reads, filtering, and denoising; (2) assigning taxonomic information to each ASV using naive Bayes classifier in QIIME 2 classifier with the 16S gene of V1-V2 region data of Greengenes to determine the identity and composition of the bacterial genera. The Shannon index, phylogenetic index and observed species were evaluated as α-diversity [35].

### 2.9. Statistical Analyses

Data were analyzed by Student’s *t* test or a one-way analysis of variance followed by Bonferroni’s multiple comparisons test, as appropriate. Bartlett’s test was used to determine the homology of variances. All statistical analyses were performed using GraphPad Prism version 6.04 (GraphPad, La Jolla, CA, USA). All tests were two-tailed, and *P* values <0.05 were considered to indicate statistical significance.

## 3. Results

### 3.1. Sevelamer Improves CDHF Diet-Induced Steatohepatitis

**Figure 1:**
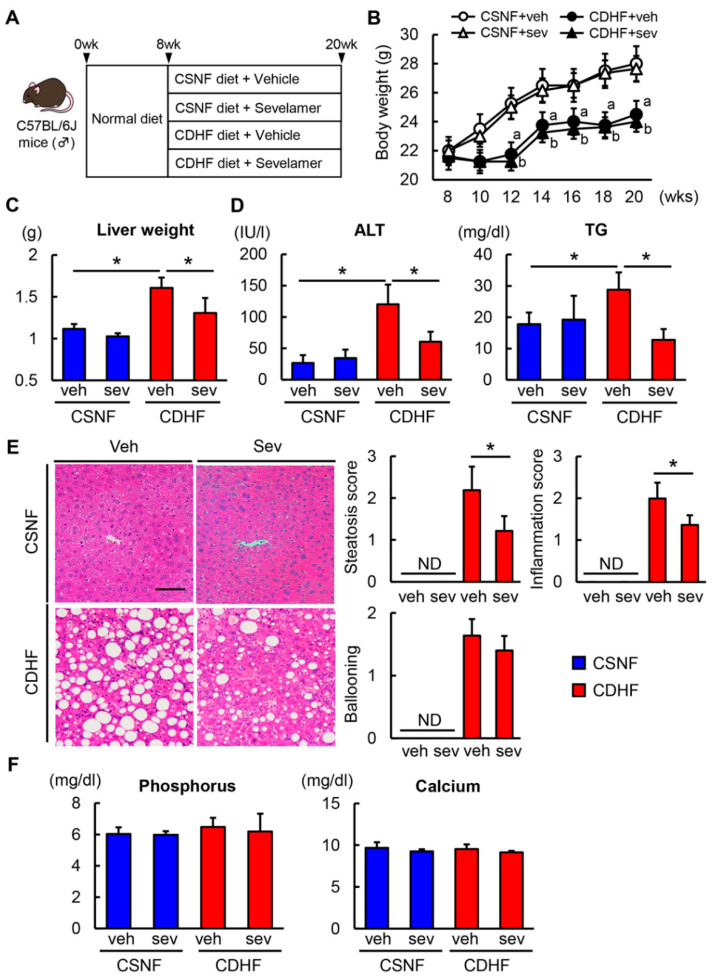
Effects of sevelamer on hepatic steatosis in choline-deficient, L-amino acid-defined, high-fat (CDHF)-fed mice. (**A**) Experimental protocols. (**B**) Changes in body weights during experimental period. (**C**) Liver weight at the end of experiment. (**D**) Serum levels of alanine aminotransferase (ALT) and triglyceride (TG). (**E**) Representative microphotographs of liver sections stained with hematoxylin–eosin (H&E) in the experimental groups. Scale Bar; 50 µm. Histological score for steatosis, inflammation and ballooning according to nonalcoholic fatty liver disease (NAFLD) Activity Score. (**F**) Serum levels of phosphorus and calcium. Vehicle (veh), sevelamer (sev), choline-sufficient amino acid containing normal fat (CSNF), choline-deficient, L-amino acid-defined containing high-fat (CDHF), not detected (ND). Data are mean ± SD (*n* = 10). * *p* < 0.05, indicating a significant difference between groups. ^a^ *p* < 0.05, indicating a significant difference compared with CSNF + veh group. ^b^ *p* < 0.05, indicating a significant difference compared with CSNF + sev group.

Experimental protocols are shown in Figure 1A. The CDHF-fed C57BL/6 mice displayed a temporary decrease in body weight in the first two weeks of the experiment, which was followed by a gradual weight gain. The CDHF-fed mice showed a lower body weight compared with the CSNF-fed control mice after the fourth week, and the sevelamer treatment did not affect the body weight of both diet-fed mice (Figure 1B). Conversely, the liver weight of the CDHF-fed mice was higher than that of the CSNF-fed mice. The sevelamer treatment did not alter the liver weight of CSNF-fed mice, while it suppressed the observed hepatomegaly in CDHF-fed mice (Figure 1C). The levels of serum alanine aminotransferase and triglycerides, which were higher in the CDHF-fed mice than in the CSNF-fed mice, were lower in those treated with sevelamer (Figure 1D). The histological evaluation of hematoxylin/eosin-stained specimens revealed severe hepatocellular steatosis, inflammation and hepatocyte ballooning in the CDHF-fed mice, whereas the sevelamer treatment led to significant improvements in steatosis and inflammatory changes and a trend for improvement in hepatocyte ballooning induced by CDHF (Figure 1E). Notably, the present dose of sevelamer did not significantly affect serum phosphorus and calcium levels in mice (Figure 1F).

**Figure 2:**
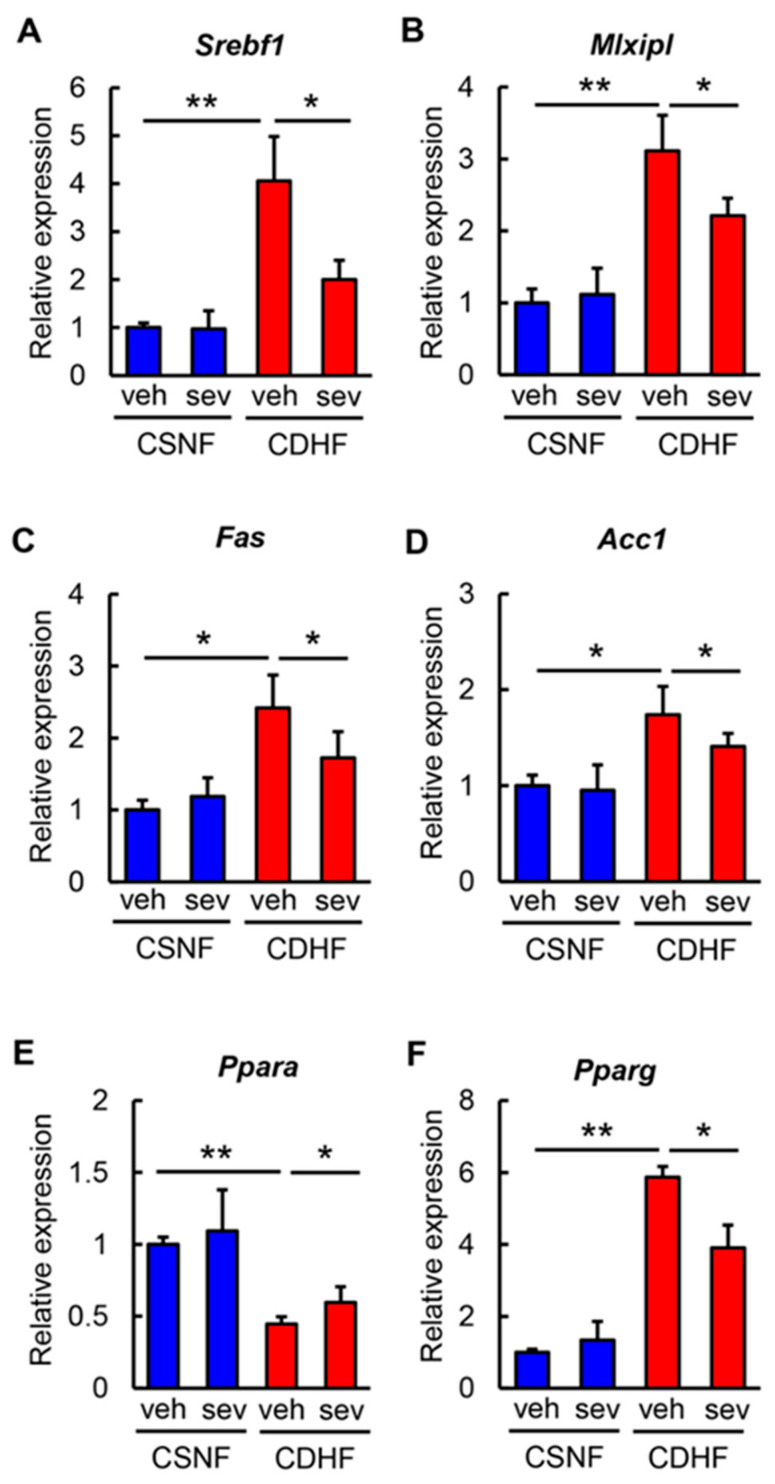
Effects of sevelamer on hepatic lipid metabolism in CDHF-fed mice. (A-F) Relative mRNA expression levels of (**A**) *Srebf1,* (**B**) *Mlxipl,* (**C**) *Fas,* (**D**) *Acc1,* (**E**) *Ppara,* and (**F**) *Pparg* in the liver of experimental groups. The mRNA expression levels were measured by quantitative RT-PCR (qRT-PCR), and 18s rRNA was used as internal control for qRT-PCR. Quantitative values are indicated as ratios to the values of CSNF + veh group. Vehicle (veh), sevelamer (sev), choline-sufficient amino acid containing normal fat (CSNF), choline-deficient, L-amino acid-defined containing high-fat (CDHF). Data are mean ± SD (*n* = 10). * *p* < 0.05, indicating a significant difference between groups. ** *p* < 0.01, indicating a significant difference between groups.

Regarding to hepatic lipid metabolism. the hepatic expression levels of lipogenesis-related genes, including *Srebf1*, *Mlxipl*, *Fas,* and *Acc1*, were increased in the CDHF-fed mice and were suppressed by the sevelamer treatment (Figure 2A–D). The analysis of fatty acid oxidation revealed that the hepatic *Ppara* mRNA level, which was significantly reduced in the CDHF-fed mice, was reversed in those treated with sevelamer (Figure 2E). Additionally, we identified that the sevelamer treatment significantly inhibited the upregulation of the adipogenic *Pparg* gene in CDHF-fed mice in parallel with the observed improvement in hepatic steatosis (Figure 2F).

### 3.2. Sevelamer Decreases Hepatic Macrophage Infiltration, Inflammatory Response, and Fibrosis in CDHF Diet-Fed Mice

**Figure 3:**
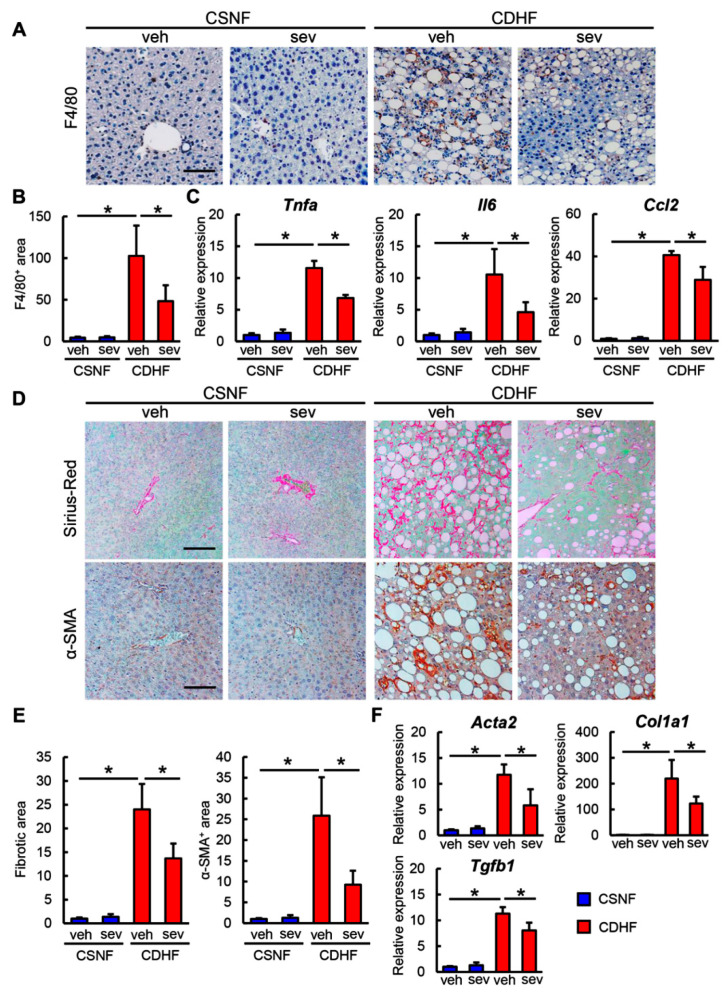
Effects of sevelamer on hepatic inflammation and fibrosis in CDHF-fed mice. (**A**) Representative microphotographs of liver sections stained with F4/80. Scale bar: 50 µm. (**B**) Semi-quantitation of F4/80 immuno-positive macrophages in high-power field (HPF) by NIH imageJ software. (**C**) Relative mRNA expression levels of proinflammatory cytokines, *Tnfa*, *Il6* and *Ccl2* in the liver of experimental groups. (**D**) Representative microphotographs of liver sections stained with Sirius Red (upper panels) and α-SMA (lower panels). Scale bar: 50 µm. (**E**) Semi-quantitation of Sirius Red-stained fibrotic area (left panel) and α-SMA immune-positive area (right panel) in HPF by NIH imageJ software. (**F**) Relative mRNA expression levels of fibrosis markers, *Col1a1*, *Acta2* and *Tgfb1* in the liver of experimental groups. (**C**,**F**) The mRNA expression levels were measured by quantitative RT-PCR (qRT-PCR), and 18s rRNA was used as internal control for qRT-PCR, and quantitative values are indicated as ratios to the values of CSNF + veh group. Vehicle (veh), sevelamer (sev), choline-sufficient amino acid containing normal fat (CSNF), choline-deficient, L-amino acid-defined containing high-fat (CDHF). Data are mean ± SD (*n* = 10). * *p* < 0.05, indicating a significant difference between groups.

Given the observed improvements in steatosis and inflammation following sevelamer treatment, we next compared the inflammatory status of mice in different experimental conditions. As shown in Figure 3A,B, we found that there was a pronounced infiltration of F4/80-positive macrophages in the livers of CDHF-fed mice and that the sevelamer treatment efficiently attenuated the hepatic macrophage infiltration in the CDHF-fed mice. In this context, the hepatic mRNA levels of proinflammatory cytokines, including *Tnfa*, *Il6*, and *Ccl2*, were substantially elevated in the CDHF-fed mice; the sevelamer treatment led to a significant suppression of these CDHF-induced changes (Figure 3C). These results indicated that sevelamer alleviated hepatic macrophage infiltration and inflammatory response in the setting of steatohepatitis.

Based on the antisteatotic and anti-inflammatory activities of sevelamer, we next assessed its effect on the development of hepatic fibrosis. As shown in Figure 3D,E, the CDHF-fed mice developed hepatic fibrosis in centrilobular areas, which was observed as pericellular fibrosis by Sirius Red staining, whereas the sevelamer treatment led to an improvement in the CDHF-mediated hepatic fibrosis. In agreement with the attenuation of fibrosis, the α-SMA-positive areas were significantly reduced in the sevelamer-treated and CDHF-fed mice compared to the vehicle-treated and CDHF-fed mice (Figure 3D,E). The observed sevelamer-mediated attenuation of hepatic fibrosis coincided with a decline in the hepatic expression levels of profibrotic genes including *Col1a1, Acta2*, and *Tgfb1* (Figure 3F).

### 3.3. Sevelamer Reduces Portal Bile Acid Levels and Inhibits the Activation of Hepatic and Intestinal FXR Signaling in CDHF Diet-Fed Mice

**Figure 4:**
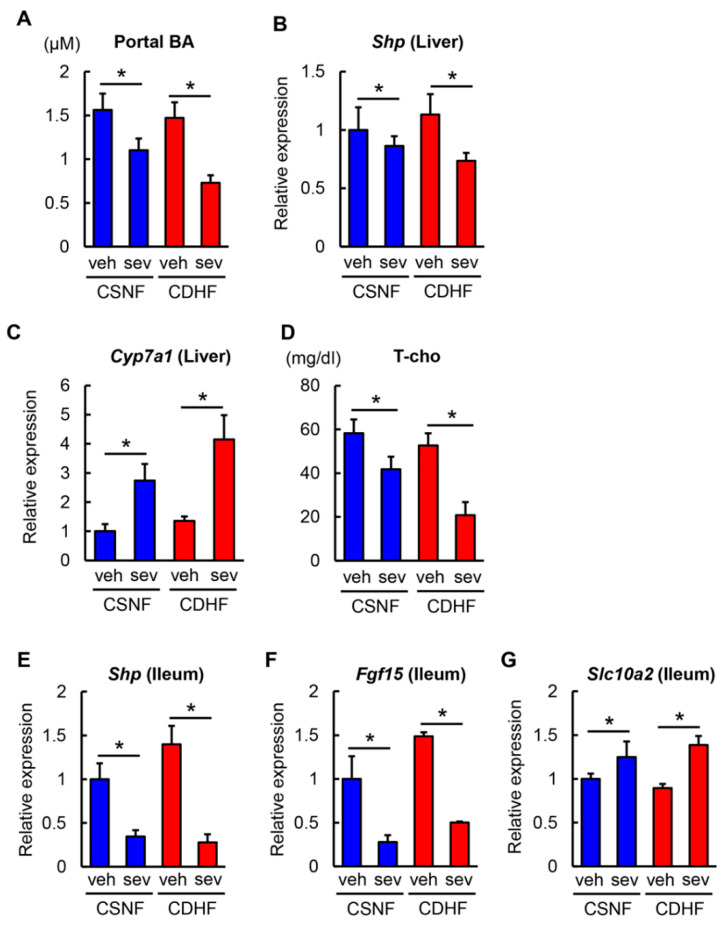
Effects of sevelamer on bile acid metabolism and hepatic and intestinal FXR signaling in CDHF-fed mice. (**A**) Total bile acid concentration in the portal vein. (**B**,**C**) Relative mRNA expression levels of *Shp* and *Cyp7a1* in the liver of experimental groups. (**D**) Serum levels of total cholesterol (T-cho). (**E**–**G**) Relative mRNA expression levels of *Shp*, *Fgf15* and *Slc10a2* in the ileum of experimental groups. (**B**,**C**,**E**–**G**) The mRNA expression levels were measured by quantitative RT–PCR (qRT-PCR), and 18s rRNA was used as internal control for qRT-PCR. Quantitative values are indicated as ratios to the values of CSNF + veh group. Vehicle (veh), sevelamer (sev), choline-sufficient amino acid containing normal fat (CSNF), choline-deficient, L-amino acid-defined containing high-fat (CDHF). Data are mean ± SD (*n* = 10). * *p* < 0.05, indicating a significant difference between groups.

To determine the mechanisms underlying the improved steatohepatitis and fibrosis observed in sevelamer-treated mice, we first investigated the enterohepatic bile acid circulation. As shown in Figure 4A, CDHF consumption did not significantly affect the serum levels of total bile acid in the portal vein, while the sevelamer treatment led to a marked reduction in the portal levels of total bile acid in both CSNF-fed and CDHF-fed mice. Bile acid synthesis is regulated by a FXR-driven negative feedback in the liver. In agreement with the decreased levels of portal bile acids, the sevelamer-treated mice exhibited a remarkable downregulation in the hepatic expression of a nuclear receptor *Shp,* and upregulation of *Cyp7a1*, the rate-limiting enzyme involved in bile acid synthesis from cholesterol (Figure 4B,C). Consequently, the serum levels of total cholesterol were also lower in the sevelamer-treated mice. (Figure 4D). These findings indicate that sevelamer-mediated reduction in portal bile acid could inhibit hepatic FXR activation and promote bile acid synthesis. On the other hand, the hepatic expression levels of *Fxr* and *Fgfr4* were unchanged in the sevelamer-treated mice (data not shown).

Next, we examined intestinal FXR signaling to evaluate whether sevelamer could prevent bile acid reabsorption. As shown in Figure 4E,F, ileal expressions of *Shp* and *Fgf15* were significantly decreased by the sevelamer treatment in both CSNF-fed and CDHF-fed mice reflecting the inhibition of intestinal FXR activation. Conversely, ileal expressions of *Slc10a2* encoding an apical sodium-dependent bile acid transporter (ASBT) were upregulated due to negative regulation by *Shp* downregulation (Figure 4G). These suggest that sevelamer might act as a bile acid sequestrant to prevent bile acid reabsorption in the gut.

### 3.4. Sevelamer Recovers the Impaired Gut Microbiota and Intestinal Barrier Function in CDHF Diet-Fed Mice

**Figure 5:**
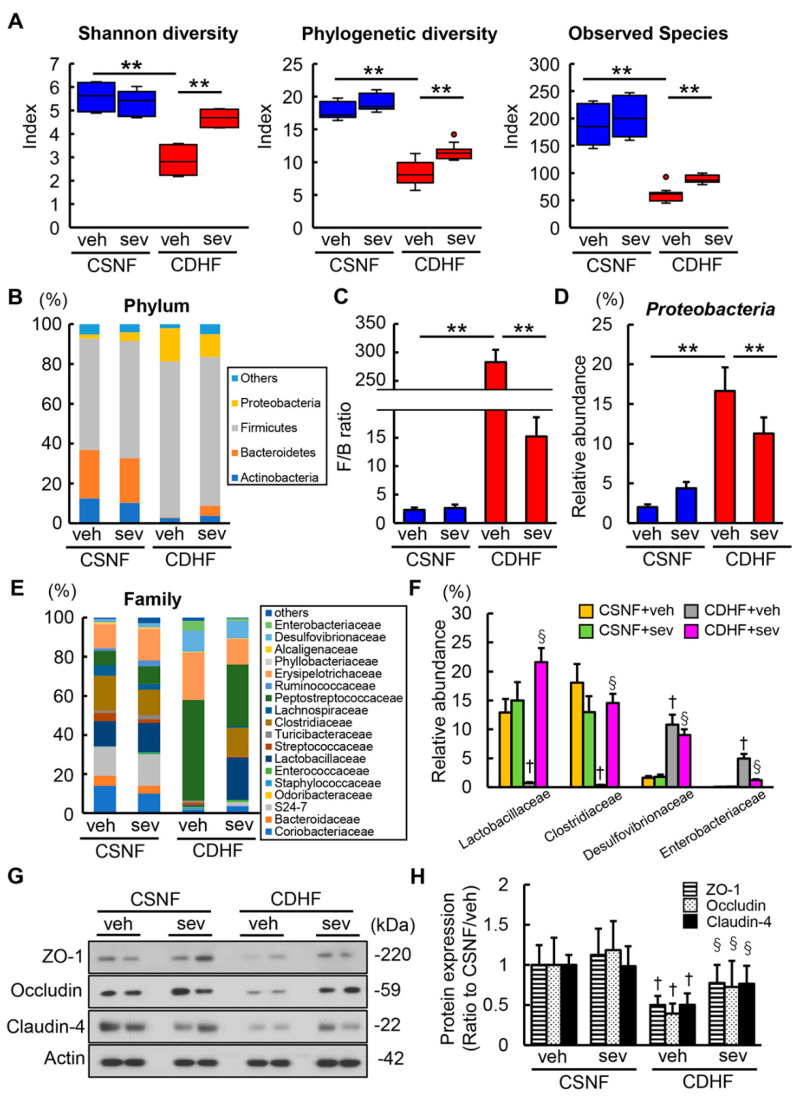
Effects of sevelamer on gut microbiota and intestinal barrier function in CDHF-fed mice. (**A**) Comparative analysis of shannon diversity, phylogenetic diversity and observed species in gut microbiota of the contexts from terminal ileum of experimental mice. (**B**) Relative abundances of predominant bacteria at the phylum levels. (**C**) The ratio of Firmicutes: Bacteroidetes (F/B) at the phylum level in the experimental groups. (**D**) The proportion of the phylum *Proteobacteria*. (**E**) Relative abundances of predominant bacteria at the family levels. (**F**) The proportion of the family *Lactobacillaceae*, *Clostridiaceae*, *Desulfovibrionaceae* and *Enterobacteriaceae*. (**G**) Western blots of intestine of experimental groups for Zo-1, Occuldin, Claudin-4. Actin was used as internal control for western blotting. Reassembly of noncontiguous gel lanes were clearly demarcated by white spaces or black lines. (**H**) Relative phosphorylation rate of Zo-1, Occludin and Claudin-4. Vehicle (veh), sevelamer (sev), choline-sufficient amino acid containing normal fat (CSNF), choline-deficient, L-amino acid-defined containing high-fat (CDHF). Data are mean ± SD (*n* = 5). ** *p* < 0.01, indicating a significant difference between groups. † *p* < 0.05, indicating a significant difference compared with CSNF + veh group. § *p* < 0.05, indicating a significant difference compared with CDHF + veh group.

Recent reports have demonstrated that sevelamer could potentially modify the impairment of gut microbiota in NASH rodent models [36,37]; therefore, we next analyzed the fecal microbiota. The global structure analysis showed a profound decrease in α-diversity (Shannon index, phylogenetic index and observed species) in the CDHF-fed mice compared with the CSNF-fed mice [35]. Interestingly, the sevelamer treatment led to an efficient recovery in the impairment of α-diversity in the CDHF-fed mice (Figure 5A). The compositions of fecal microbiota were also markedly altered in the CDHF-fed mice. In line with the previous report, CDHF-fed mice showed an significantly higher ratio of *Firmicutes*: *Bacteroidetes* at the phylum level, the gut microbial feature related to metabolic syndrome (Figure 5B,C) [38]. Moreover, CDHF consumption significantly increased the proportion of the phylum *Proteobacteria*, mainly composed of gram-negative bacteria (Figure 5D). Remarkably, these changes of microbial compositions in CDHF-fed mice were prevented by treatment with sevelamer (Figure 5B–D). At the family level, the feces of CDHF-fed mice displayed decreases in *Lactobacillaceae* and *Clostridiaceae* producing lactate and short chain fatty acids (SCFAs) respectively and increases in *Desulfovibrionaceae* and *Enterobacteriaceae*, which were improved by the sevelamer treatment (Figure 5E,F). Meanwhile, the sevelamer treatment did not make significant changes in the microbial diversity and composition in the CSNF-fed mice (Figure 5B–F). Moreover, CDHF led to significant decreases in the intestinal levels of tight junction proteins (TJPs) including ZO-1, occludin, and claudin-4, whereas the sevelamer treatment led to an effective improvement in the CDHF-induced loss of TJPs (Figure 5G,H).

### 3.5. Sevelamer-Mediated Reduction in LPS Contributes to Suppressing HSC Activation

**Figure 6:**
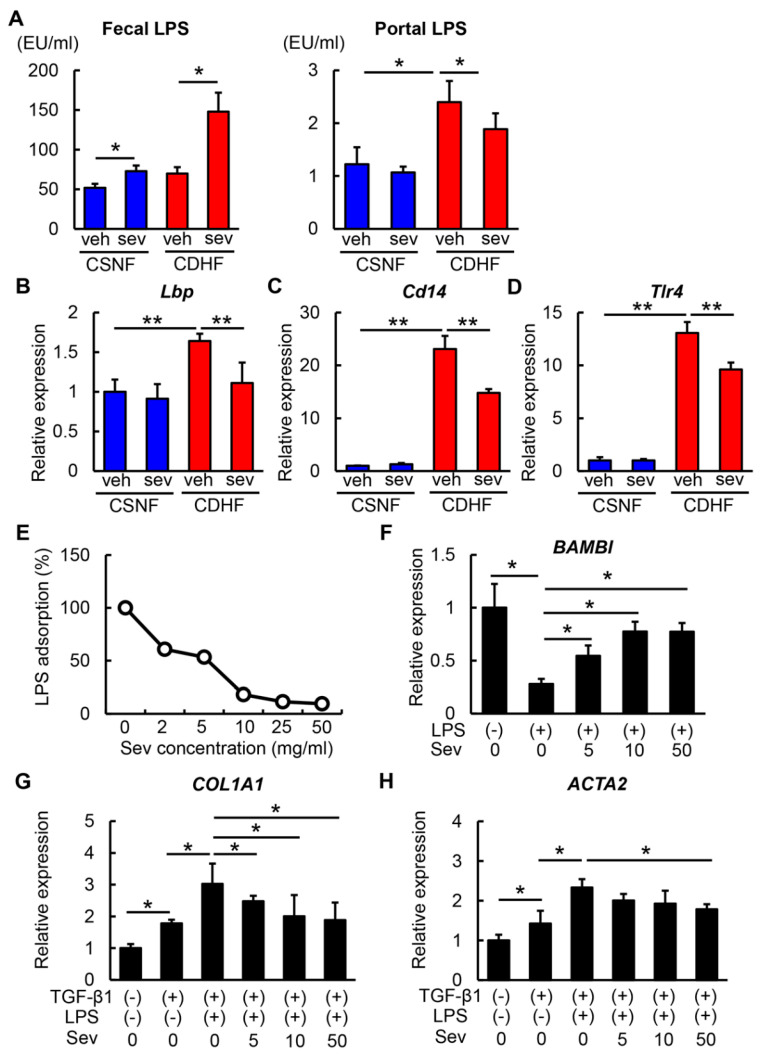
Effects of sevelamer on hepatic LPS/TLR4 signaling and in vitro hepatic stellate cell activation. (**A**) Fecal and portal LPS levels in the experimental groups. (**B**–**D**) Relative mRNA expression levels of *Lbp*, *Cd14* and *Tlr4* in the liver of experimental groups. (**E**) Dose-dependent effect of sevelamer on LPS-adsorbable action. Sevelamer (0–50 mg/mL) was incubated with LPS (100 ng/mL) for 1 h, and supernatants were then assayed for endotoxin. (**F**) Relative mRNA expression levels of *BAMBI* in LX-2 incubated with LPS (100 ng/mL) and different concentrations of sevelamer (0–50 mg/mL). (**G**,**H**) Relative mRNA expression of *COL1A1* (**G**) and *ACTA2* (**H**) in LX-2 incubated with LPS (100 ng/mL) and different concentrations of sevelamer (0–50 mg/mL) followed by preincubation with TGF-β1. (**B**–**D**,**F**–**H**) The mRNA expression levels were measured by quantitative RT-PCR (qRT-PCR), and 18s rRNA was used as internal control for qRT-PCR. Vehicle (veh), sevelamer (sev), choline-sufficient amino acid containing normal fat (CSNF), choline-deficient, L-amino acid-defined containing high-fat (CDHF). Data are mean ± SD ((**B**–**D**): *n* = 10, (**F**–**H**): *n* = 6). * *p* < 0.05, indicating a significant difference between groups.

We next focused on the LPS-binding affinity of sevelamer. To evaluate whether sevelamer adsorbed LPS in the intestines, we measured fecal and portal LPS concentrations. As shown in Figure 6A, sevelamer treatment provided mild elevation of fecal LPS levels and decline of portal LPS levels in the CSNF-fed mice. Remarkably, the changes in the fecal and portal LPS levels by sevelamer treatment conspicuously appeared in the CDHF-fed mice. These findings suggested that sevelamer could enhance the fecal excretion of LPS potentially by LPS adsorption.

In accordance with restored intestinal TJPs and promoted fecal LPS excretion, sevelamer treatment lead to the suppression of the upregulated hepatic expression of *Lbp* (LPS-binding protein) that binds to LPS to form a complex, which interacts with the macrophage receptor to initiate the proinflammatory host response in CDHF-fed mice (Figure 6B). The mRNA levels of *Cd14* and toll-like receptor 4 (*Tlr4*), a coreceptor that functions with TLR4 to detect LPS, were overexpressed in the livers of CDHF-fed mice, whereas the sevelamer treatment effectively inhibited the overexpression of *Cd14* and *Tlr4* in the CDHF-fed mice (Figure 6C,D).

Subsequently, we assessed the impact of sevelamer-mediated reduction in hepatic LPS on fibrogenic activity in the LX-2 human HSC line. We confirmed that sevelamer led to a dose-dependent reduction in the LPS levels in cultured media, reflecting its LPS-adsorbing activity (Figure 6E). In accordance with the known evidence, LPS significantly reduced the mRNA expression level of bone morphogenetic protein and activin membrane-bound inhibitor (*Bambi*), a TGF-β pseudoreceptor, in LX-2 cells [39]. Interestingly, in LX-2 cells the sevelamer treatment attenuated the LPS-mediated reduction in *BAMBI* expression in a dose-dependent manner (Figure 6F). Furthermore, LPS augmented the TGF-β1-mediated induction of *COL1A1* and *ACTA2*, which was attenuated by the sevelamer treatment, consistent with the increased *BAMBI* expression level (Figure 6G,H).

## 4. Discussion

In the present study, we demonstrated that sevelamer, the bile acid-sequestering resin improved the CDHF-induced hepatic steatosis, inflammation, and fibrosis by reducing hepatic overload of LPS due to rearrangement of dysbiosis and intestinal LPS adsorption as well as inhibition of bile acid reabsorption. We found that sevelamer-mediated reductions in both the hepatic and ileal expression levels of the FXR target gene *Shp* in CDHF-fed mice, indicating that sevelamer pharmacologically reduced the bile acid reabsorption in terminal ileum by binding with bile acids in the intestinal tract. This is in agreement with the findings in recent report from McGettigan et al. showing that sevelamer-mediated bile acid sequestration ameliorates liver steatosis and inflammation by inhibiting the activation of hepatic and intestinal FXR signaling in WD-fed mice [29]. Currently, whether and how the activation of FXR affects the progression of NASH is controversial. Several lines of evidence have supported that hepatic FXR activation can potentially attenuate steatohepatitis based on the beneficial outcomes observed in patients with NASH treated with FXR agonists [40,41,42], whereas intestinal FXR activation has been reported to promote NAFLD pathology [43]. Moreover, it has recently been reported that the hepatoprotective effect with ASBT inhibitor-mediated inhibition of bile acid reabsorption was limited in the CDAA-induced NASH model [44]. These lines of evidence led us to assess additional mechanisms which might be related to the observed suppressive effects of sevelamer in NASH progression in mice.

Our data regarding fecal microbiome showed that the CDHF-fed mice also displayed a significantly lower α-diversity defined by decreases in Shannon index, phylogenetic index and observed species, which was suppressed by sevelamer treatment [35]. Importantly, the analysis of the overall composition revealed that sevelamer significantly decreased the relative abundance of LPS-producing gram-negative phylum *Proteobacteria* including the families of *Desulfovibrionaceae* and *Enterobacteriaceae* in the CDHF-fed mice. We also found that sevelamer treatment increased *Lactobacillaceae* and *Clostridiaceae* producing lactate and SCFAs respectively which are recognized to protect intestinal barrier function [45,46]. Altogether, these results suggest that sevelamer-mediated microbial modification is closely associated with a decrease in portal LPS levels accompanied with the restoration of intestinal levels of TJPs in the CDHF-fed mice. Additionally, we evaluated the LPS-binding affinity of sevelamer. Recent studies have provided direct evidence that sevelamer exhibits in vitro LPS-binding properties [30,47]. Indirect clinical evidence suggests that sevelamer might also contribute to the blockade of LPS translocation from the intestinal tract to the portal circulation [48]. Coincident with these pharmacological aspects, sevelamer promoted the fecal excretion of LPS, resulting in its removal from the intestinal lumen in the present study. Thus, we propose that the removal of LPS by intestinal adsorption also participates in the sevelamer-mediated improvement in TJPs as well as above microbial modification.

Through these mechanisms, the sevelamer-mediated reduction in LPS influx to liver consequently led to the suppression of hepatic TLR4 activation. Besides the activation in macrophages, the LPS/TLR4 signaling plays a crucial role in HSC activation. Seki et al. have identified that the LPS/TLR4 signaling induces the downregulation of Bambi, a transmembrane suppressor of TGF-*β* signaling in HSCs [39]. Notably, our in vitro data have revealed that sevelamer-mediated LPS adsorption remarkably attenuates LPS-stimulated fibrogenic activity, together with the downregulation of Bambi in human HSCs. These sequential findings suggest that the inhibitory effects of sevelamer on the progression of NASH in this murine model might be due to its capacity to regulate intestinal LPS in addition to its role in bile acid sequestration.

This study has several considerable limitations. First, the bile acid composition of patients with NASH often differ from that of healthy subjects [49]. The present study results revealed that sevelamer reduced the portal levels of bile acids, whereas it remains unclear if sevelamer also alters the bile acid composition. The recent study on WD-fed mice has revealed that sevelamer alters individual bile acid species, including an increase in lithocholic acid in the ileum and decreases in most other bile acids [36]. Moreover, another report has shown that chenodeoxycholic acid, which was increased in the cecum of WD-fed mice, induced intestinal hyperpermeability, and sevelamer protected against CDCA-induced barrier dysfunction [37]. Thus, additional analyses are necessary to determine if similar changes occur following sevelamer treatment in CDHF-fed mice. Second, a recent report has suggested that LPS reverses HSC activation via the downregulation of cMyb, a transcription factor for α-SMA, and via the upregulation of SMAD7 and C/EBPδ, two transcriptional inhibitors of Col1α1 [50]. These findings partially conflict with the LPS-mediated induction of TGF-β signaling which occurs via the activation of TLR4 pathway in HSCs, although this unique action of LPS also deserves future investigation. Third, the present study does not approach to the antidiabetic effect of sevelamer because the CDHF-fed mice do not basically display typical diabetic status including insulin resistance. Recent clinical study has shown that sevelamer reduced plasma glucose concentrations in patients with type 2 diabetes and suggested that its mechanisms were possibly involved in decreased intestinal and hepatic bile acid-mediated FXR activation [51]. Therefore, additional work is needed to confirm these mechanisms by using diabetic animal models.

Finally, the current study lacks the analysis of female mice. Sex difference is a definite feature of NAFLD, and sexual dimorphism in NAFLD is increasingly recognized [52]. Indeed, a recent large cohort study showed that decreased choline intake is significantly associated with increased NASH fibrosis, which was remarkable in postmenopausal women [53]. Moreover, the response to the LPS inflammation challenge is reportedly affected by mouse sex differences [54]. These lines of evidence require further investigation to evaluate whether sevelamer shows a similar effect in CDHF-fed female mice.

Collectively, our findings demonstrate that sevelamer inhibits the development of steatohepatitis in a murine model. Several basic studies have also revealed similar effects of sevelamer on NASH. However, to the best of our knowledge, this is the first report identifying the suppressive effects of sevelamer on the hepatic overload of endogenous LPS as well as its ability to sequester bile acids as the underlying mechanisms during the development of NASH-based liver fibrosis. We emphasize that these actions of sevelamer were achieved at a pharmacological dose in the absence of adverse effects such as hypophosphatemia. To clinically apply these experimental findings, further translational research is required in the future.

## Figures and Tables

**Table 1:** List of primers for q-PCR.

Gene	Sense (5′–3′)	Antisense (5′–3′)
Mouse		
*Srebf1*	GGAGCCATGGATTGCACATT	GGCCCGGGAAGTCACTGT
*Mlxipl*	CTGGGGACCTAAACAGGAGC	GAAGCCACCCTATAGCTCCC
*Fas*	TGGGTTCTAGCCAGCAGAGT	ACCACCAGAGACCGTTATGC
*Acc1*	GCCTCTTCCTGACAAACGAG	TGACTGCCGAAACATCTCTG
*Ppara*	GAGGGTTGAGCTCAGTCAGG	GGTCACCTACGAGTGGCATT
*Pparg*	CTGTGAGACCAACAGCCTGA	AATGCGAGTGGTCTTCCATC
*Tnfa*	ACGGCATGGATCTCAAAGAC	AGATAGCAAATCGGCTGACG
*Il6*	GTTCTCTGGGAAATCGTGGA	TGTACTCCAGGTAGCTATGG
*Ccl2*	AGGTCCCTGTCATGCTTCTG	TCTGGACCCATTCCTTCTTG
*Col1a1*	GAGCGGAGAGTACTGGATCG	GCTTCTTTTCCTTGGGGTTC
*Acta2*	CTGACAGAGGCACCACTGAA	CATCTCCAGAGTCCAGCACA
*Tgfb1*	TTGCTTCAGCTCCACAGAGA	TGGTTGTAGAGGGCAAGGAC
*Shp*	CGATCCTCTTCAACCCAGATG	AGGGCTCCAAGACTTCACACA
*Cyp7a1*	AGCAACTAAACAACCTGCCAGTACTA	GTCCGGATATTCAAGGATGCA
*Fxr*	GTTGCCGTGAGGAAGCTAAG	GAACTTGAGGAAACGGGACA
*Fgf15*	GGCAAGATATACGGGCTGAT	GATGGTGCTTCATGGATCTG
*Slc10a2*	TGGGTTTCTTCCTGGCTAGACT	TGTTCTGCATTCCAGTTTCCAA
*Lbp*	GGCTGCTGAATCTCTTCCAC	GAGCGGTGATTCCGATTAAA
*Cd14*	GTCAGGAACTCTGGCTTTGC	TGGCTTTTACCCACTGAACC
*Tlr4*	GGCAGCAGGTGGAATTGTAT	AGGCCCCAGAGTTTTGTTCT
*18S rRNA*	TTGACGGAAGGGCACCACCAG	GCACCACCACCCACGGAATCG
Human		
*BAMBI*	GGCAGCATCACAGTAGCATC	GATCGCCACTCCAGCTACAT
*COL1A1*	CCAAATCTGTCTCCCCAGAA	TCAAAAACGAAGGGGAGATG
*ACTA2*	GAGACCCTGTTCCAGCCATC	TACATAGTGGTGCCCCCTGA
*18S rRNA*	AAACGGCTACCACATCCAAG	CCTCCAATGGATCCTCGTTA

## Supplementary Materials

The following are available online: https://www.mdpi.com/2076-2607/8/6/925/s1, Table S1: List of diet components.
