# Supplementary material for: Bile Acid Sequestrant, Sevelamer Ameliorates Hepatic Fibrosis with Reduced Overload of Endogenous Lipopolysaccharide in Experimental Nonalcoholic Steatohepatitis
Source: Microorganisms. 2020 Jun 19;8(6):925. doi: 10.3390/microorganisms8060925 (PMC7357162; doi:10.3390/microorganisms8060925)
Supplement: Supplementary file 1 [file microorganisms-08-00925-s001.pdf]

Supplementary Table 1. List of diet component.

	CDHF	CSNF
Protein (kcal%)	18	18
Carbohydrate (kcal%)	20	71
Fat (kcal%)	62	10
Protein (gm%)	23.1	17.2
Carbohydrate (gm%)	26.3	67.4
Fat (gm%)	35.7	4.3
Fiber (gm%)	6.6	4.8
Kcal/gm	5.2	3.8
Casein, 80 Mesh (gm)	0	0
L-Cystine (gm)	4.2	4.2
L-Isoleucine (gm)	7.6	7.6
L-Leucine (gm)	15.8	15.8
L-Lysine (gm)	13.2	13.2
L-Methionine (gm)	0.8	5.1
L-Phenylalanine (gm)	8.4	8.4
L-Threonine (gm)	7.2	7.2
L-tryptophan (gm)	2.1	2.1
L-Valine (gm)	9.3	9.3
L-Histidine (gm)	4.6	4.6
L-Alanine (gm)	5.1	5.1

L-Arginine (gm)	6.0	6.0
L-Aapartic Acid (gm)	12.1	12.1
L-Glutamic Acid (gm)	38.2	38.2
Glycine (gm)	3.0	3.0
L-Proline (gm)	17.8	17.8
L-Serine (gm)	10.0	10.0
L-Tyrosine (gm)	9.2	9,2
Corn Starch (gm)	0	502
Maltodextrin 10 (gm)	130.1	130.1
Sucrose (gm)	68.8	68.8
Cellulose, BW200 (gm)	50	50
Soybean Oil (gm)	25	25
Lard (gm)	245	20
Mineral Mix S10026 (gm)	10	10
DiCalcium Phosphate (gm)	13	13
Calcium Carbonate (gm)	5.5	5.5
Potassium Citrate (gm)	16.5	16.5
Sodium BiCarbonate (gm)	7.5	7.5
Vitamine Mix V10001 (gm)	10	10
Choline Bitartrate (gm)	0	2
